# Conservation Priorities Analysis of Chinese Indigenous Pig Breeds in the Taihu Lake Region

**DOI:** 10.3389/fgene.2021.558873

**Published:** 2021-03-03

**Authors:** Qing-bo Zhao, Eugenio López-Cortegano, Favour Oluwapelumi Oyelami, Zhe Zhang, Pei-pei Ma, Qi-shan Wang, Yu-chun Pan

**Affiliations:** ^1^Department of Animal Science, School of Agriculture and Biology, Shanghai Jiao Tong University, Shanghai, China; ^2^Institute of Evolutionary Biology, School of Biological Sciences, University of Edinburgh, Edinburgh, United Kingdom; ^3^Department of Animal Breeding and Reproduction, College of Animal Science, Zhejiang University, Hangzhou, China

**Keywords:** Chinese indigenous pig, conservation priority, gene diversity, allelic diversity, meta-population

## Abstract

Most indigenous pig resources are known to originate from China. Thus, establishing conservation priorities for these local breeds is very essential, especially in the case of limited conservation funds. Therefore, in this study, we analyzed 445 individuals belonging to six indigenous breeds from the Taihu Lake Region, using a total of 131,300 SNPs. In order to determine the long-term guidelines for the management of these breeds, we analyzed the level of diversity in the metapopulation following a partition of diversity within and between breed subpopulations, using both measures of genic and allelic diversity. From the study, we found that the middle Meishan (MMS) pig population contributes the most (22%) to the total gene diversity while the Jiaxing black (JX) pig population contributes the most (27%) to the gene diversity between subpopulations. Most importantly, when we consider one breed is removed from the meta-population, the first two breeds prioritized should be JX pig breed and Fengjing pig breed followed by small Meishan (SMS), Mizhu (MI), and Erhualian (EH) if we pay more attention to the gene diversity between subpopulations. However, if the priority focus is on the total gene diversity, then the first breed to be prioritized would be the Shawutou (SW) pig breed followed by JX, MI, EH, and Fengjing (FJ). Furthermore, we noted that if conservation priority is to be based on the allelic diversity between subpopulations, then the MI breed should be the most prioritized breed followed by SW, Erhuanlian, and MMS. Summarily, our data show that different breeds have different contributions to the gene and allelic diversity within subpopulations as well as between subpopulations. Our study provides a basis for setting conservation priorities for indigenous pig breeds with a focus on different priority criteria.

## Introduction

Indigenous breeds are well adapted to their specific environmental conditions, and their interest in livestock is increasingly being recognized (Kim et al., 2019; Lukić et al., 2020). However, it is difficult or almost impossible to protect all the livestock species in the world. Therefore, determining conservation priorities is key to their effective management. Conservation priorities have been previously established using measures of genic or allelic diversity for different livestock species using a variety of genomic markers. For example, Fabuel et al. (2004) analyzed the conservation priority in Iberian pigs based on microsatellite markers, Ramljak et al. (2018) performed the conservation of a domestic metapopulation by using SNP chips of 60 different cattle breeds, and Zhang et al. (2019) analyzed the conservation priority for Chinese domestic duck breeds by using the short tandem repeat profiling. However, no systematic assessment for the conservation priority of the Chinese indigenous pig breeds has been conducted. Therefore, to effectively protect the indigenous pig genetic resources and to ensure the sustainable development of the pig industry in China, it is necessary to determine their conservation priorities, especially when the conservation funding is limited.

The Taihu pig breed, which originates from the Taihu Lake region, is regarded among the world’s most prolific breeds with many excellent characteristics such as desirable meat quality, high resistance to disease, and good adaptability to the local environment (Duchet-Suchaux et al., 1991; Li et al., 2013; Ma et al., 2013). They include six breeds: Erhualian (EH), Meishan (MS), Fengjing (FJ), Jiaxing black (JX), Mizhu (MI), Shawutou (SW), and Hengjing (a breed presently in extinction). The MS breed was further divided into two strains called the middle Meishan (MMS) and the small Meishan (SMS) (Zhang, 1991). Therefore, in this study, we used the Taihu pig breeds as a case study to analyze the conservation priority of Chinese indigenous pig breeds and to provide a basis to better allocate funds for breed insurance.

Weitzman (1992, 1993) proposed a popular theory of diversity to prioritize populations for conservation using a rational framework and this method has been accepted in conservation studies of domestic animals (Canon et al., 2001; Reist-Marti et al., 2003; Lenstra, 2006; Tapio et al., 2006). Moreover, some methods that have been designed using molecular kinships have also been used to evaluate the value of making conservation decisions in European cattle breeds (Eding et al., 2002; Mateus et al., 2004; Bennewitz et al., 2006; Medugorac et al., 2011). In this study, we performed an analysis on conservation priorities based on gene and allelic diversity by considering the intended purpose of conservation, including short-term or long-term goals.

## Materials and Methods

**FIGURE 1:**
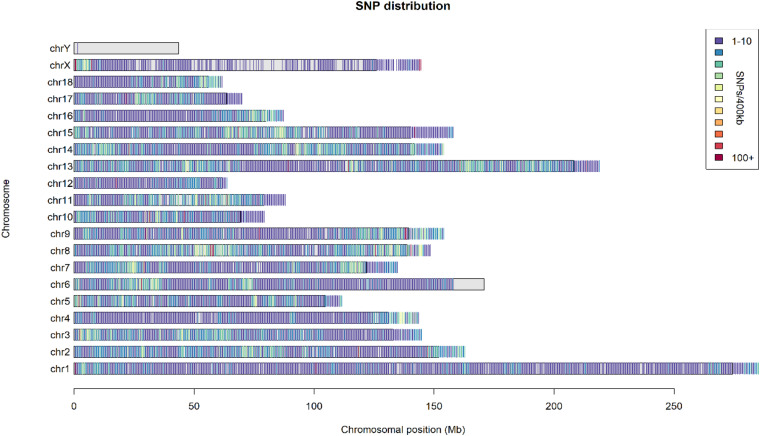
The distribution of SNPs on each chromosome. The different colors in the legend represent SNP density per 400 kb.

A total of 445 unrelated pigs (75 SMS, 97 medium Meishan, 36 MI, 42 EH, 91 JX, 72 SW, and 32 FJ) from the seven Chinese indigenous populations in the Taihu Lake region were selected (Zhao et al., 2019). Then, DNA samples were extracted from ear tissue. After sequencing, filtering, and imputation, a total of 131,300 SNPs with MAF ≥ 0.05 were used to do the further analysis. The distribution of SNPs on each chromosome was relatively uniform except for the Y chromosome (Figure 1).

Population structure based on all SNPs was determined using the principal component analysis (PCA) and *t*-distributed stochastic neighbor embedding (*t*-SNE) (van der Maaten and Hinton, 2008). The eigenvectors for each individual were calculated by using the GCTA software version 1.91.6 (Yang et al., 2011).

Total genic diversity $\left(H_{T}=1-\bar{f}\right)$ in a subdivided population can be divided into two components as $H_{T}=\left(1-\tilde{f}\right)+D_{G}=H_{S}+D_{G}$ following Nei (1973), where $\bar{f}$ is the kinship or average co-ancestry among all individuals of the population, $\tilde{f}$ is the average of within-subpopulation co-ancestries, H_*S*_ refers to the within-subpopulations genic diversity, and *D*_*G*_ is the average Nei’s genetic distance between subpopulations. Furthermore, H_*S*_ can be divided into two components: the genic diversity within individuals, and the genetic diversity between individuals.

We determined the contribution of every subpopulation to the total gene diversity, as well as its components by decomposing the two terms of the following expression for every subpopulation: $H_{T}=\left(1-\tilde{f}\right)+D_{G}=H_{S}+D_{G}$, as described above. Moreover, we utilized an analogous partition to estimate the allelic diversity from the following expression in the study of Caballero and Toro (2002) and Caballero et al. (2010):

$$A_{T}=A_{S}+D_{A}=\left[\frac{1}{n}\,\sum_{i=1}^{n}\left(a_{i}\,\frac{1}{n}\,\sum_{j=1}^{n}d_{i,j}\right)\right]-1$$

Where *a*_*i*_ is the expected number of different alleles taken at random in a sample of genes, and *d*_*i,j*_ is the average allelic distance of subpopulations i and *j*. The software Metapop2 v2.3 (López-Cortegano et al., 2019a) was used to compute these components of genic and allelic diversity within and between subpopulations, as well as the expected contribution of each breed to the metapopulation diversity. This was done by estimating the change in diversity after a population is removed, following Petit et al. (1998). In addition, Metapop2 was also used to compute each breed contribution to a synthetic pool of size *N* = 1,000, in order to maximize either gene or allelic diversity. Under this method, the relative contributions of each subpopulation to the synthetic pool are given at random, and maximized throughout iterations until maximum genetic diversity (genic or allelic) is reached, using a simulated annealing algorithm (Kirkpatrick et al., 1983). Rarefaction was enabled to correct allelic diversity measures by population size.

Allele frequency data was also collected from Metapop2 output, and used to search alleles private to each breed. For every private allele, genomic coordinates were taken, and used to query the corresponding loci and functional annotation, if available.

## Results

**FIGURE 2:**
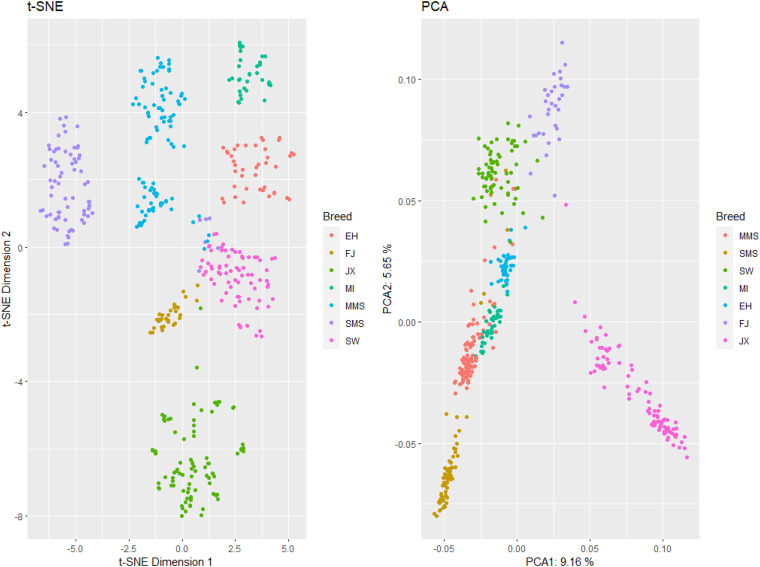
Left: *t*-SNE plot (dimension 1 and dimension 2); right: PCA plot (PCA1 and PCA2). Breeds are represented in colors. SMS, small Meishan pig; MMS, middle Meishan pig; SW, Shawutou pig; MI, Mizhu pig; EH, Erhualian pig; FJ, Fengjing pig; JX, Jiaxing black pig.

The result of our population structure analysis using PCA and *t*-SNE method showed that the seven studied populations are well separated, including the MMS and SMS subpopulations. Overall, the most differentiated breed was JX (Figure 2). A 9.16% and 5.65% of the total variation is explained by the first and second principal components.

**FIGURE 3:**
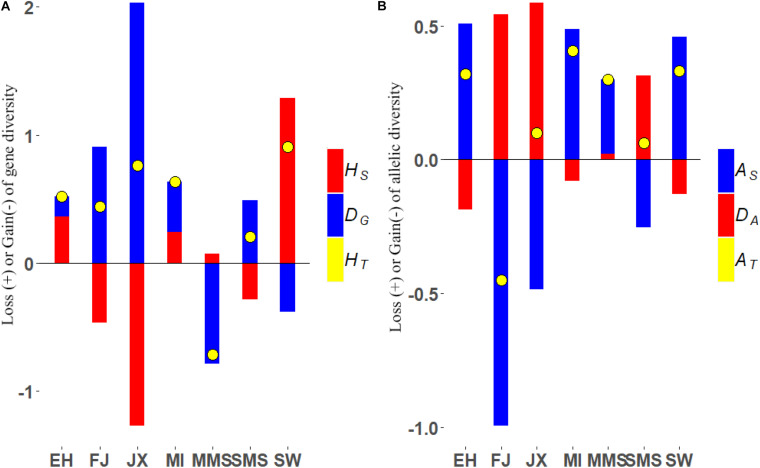
**(A)** Loss (+) or gain (−) of gene diversity after removal of each subpopulation (%). H_*S*_, gene diversity within subpopulations; D_*G*_, gene diversity between subpopulations; H_*T*_, the total loss or gain gene diversity. **(B)** Loss (+) or gain (−) of allelic diversity after removal of each subpopulation (%). A_*S*_, allelic diversity within subpopulations; D_*A*_, allelic diversity between subpopulations; A_*T*_, the total loss or gain allelic diversity;

The loss and gain of gene diversity and allelic diversity after removal of each subpopulation is shown in Figure 3 and Supplementary Tables 2, 3. Regarding the total gene diversity, we found that the removal of SW breed caused the largest reduction of about 0.906% followed by JX (0.762%), MI (0.635%), EH (0.517%), FJ (0.438%), and SMS (0.205%). Interestingly, most contributions to H_*T*_ from the SW breed were from its within-subpopulation diversity component (as it was the less inbred strain, with *F* = 0.63, and also with a strongly negative deviation from Hardy–Weinberg equilibrium, see Supplementary Table 4), while JX contribution was mainly from D_*G*_, since it is the most differentiated breed. In fact, among all the breeds, JX displayed the highest amount of contribution to between-subpopulations diversity (2.032%, Figure 3A), followed by FJ (0.907%), SMS (0.491%), MI (0.396%), and EH (0.154%). However, the conservation priority would be changed if we focus more on the gene diversity between individuals and the EH breed would be the most prioritized breed followed by JX, MI, and MMS. In general, we observed that the H_*T*_ was constantly reduced after the removal of any of the breeds from the metapopulation, with the exception of MMS, given its low degree of genetic differentiation when compared to other breeds.

Regarding allelic diversity, the contributions to total diversity generally ranked similarly between breeds as for genic diversity, with the notable exception of MMS, which ranked 4th among the breeds that contributed positively to allelic richness (A_*S*_) and exhibited a certain degree of allelic differentiation. Another exception was the FJ breed, which contributed negatively to A_*T*_ because of its low A_*S*_ and positively to H_*T*_ because of its high degree of genic differentiation. Of a note, among all the studied breeds, the MI subpopulation contributed the most to the total allelic diversity, leading to the largest reduction in diversity when removed (0.407%), especially due to its large contribution to A_*S*_ (0.487%). Moreover, based on this priority, we found that the SW (0.332%), EH (0.319%), and MMS (0.301%) ranked next to MI, respectively. Further results showed that JX was still the most differentiated breed, with the highest contribution to the between-subpopulations allelic diversity component (0.586%), followed by FJ (0.543%), SMS (0.315%), and MMS (0.021%). Regarding allelic differentiation, it is worth mentioning that only the JX and MMS breeds retained private alleles, suggesting that gene flow has been more limited to and from these populations.

In particular, a total of 111 private alleles were found for JX, while 10 were segregated in MMS, four in SMS and only two in SW breed (see Supplementary Table 6). However, functional analysis showed that only JX and MMS contained private alleles located in genes. There were seven private alleles in the JX breed and one in the MMS breed with a location in the protein-coding regions of genes. Both JX and MMS showed private alleles for loci associated with growing factors. For example, a private allele for the growing factor FGF14 gene, which is related to anatomical structure and development, was found in MMS, while the JX had private alleles for the bone morphogenic protein BMP5, and the CDH13 locus that is related to cell proliferation, and might be a genomic imprint in JX breeds that have undergone a long-term selection. Interestingly, the frequency of private alleles was relatively higher (0.38 + −0.08) and stable among these breeds (0.39 + −0.08 in JX, 0.30 + −0.04 in MMS, 0.37 + −0.05 in SMS, and 0.35 + −0.03 in SW), suggesting that their polymorphism may be important in the population, and is perhaps maintained by mechanisms such as balancing selection. The contributions of each subpopulation to the total gene diversity are presented in Supplementary Table 1. We found that the MMS population had the highest contribution to total gene diversity (22%) as well as to gene diversity within individuals, between individuals, and within subpopulations, whereas the EH population had the lowest contribution to total gene diversity (10%). The total allelic diversity (0.964) was mainly dependent on the component of diversity within subpopulations (0.890). Thus, we can conclude that the differences in allelic diversity among the seven pig populations of the Taihu Lake region arose mainly from the variation between individuals within the subpopulation rather than the variation between subpopulations.

**FIGURE 4:**
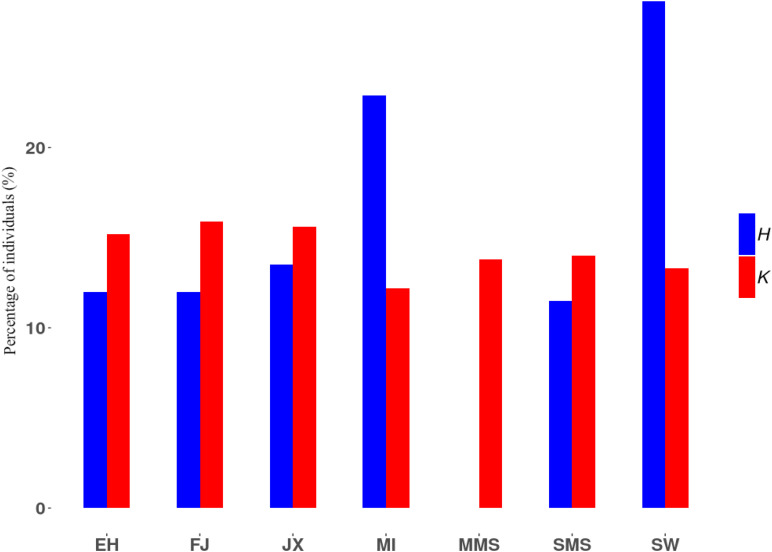
Percentage of individuals contributed from each subpopulation to a pool of individuals with maximal heterozygosity or number of alleles. H, maximal heterozygosity; K, maximal number of alleles.

Finally, we followed an alternative method to compute subpopulation contributions to a synthetic pool of *N* = 1,000 individuals, to explore the proportion of these breeds that maximize the expected heterozygosity and total number of alleles. As shown in Figure 4, maximum allelic diversity can be obtained with balanced contributions among breeds. However, maximum genic diversity requires a larger contribution of SW and MI breeds, while MMS individuals should be excluded from the pool, in agreement with its negative contribution to H_*T*_ as shown above.

In this study, we focused on conservation priorities for Chinese indigenous pig populations in the Taihu Lake region, with the aim of assessing their values to make reasonable conservation decisions and better allocate conservation funds. From our results, different populations show different contributions to the gene diversity and allelic diversity within subpopulations as well as between subpopulations. For example, if we focus on total gene diversity, the SW breed should be the first conservation priority. However, if the focus is on total allelic diversity, then the MI breed will be the first conservation priority. In conclusion, to better manage animal genetic resources, different conservation priorities should be set based on different focus or conservation purpose.

## Discussion

The main purpose of conservation programs is to maintain a high level of gene and allelic diversity within one population. In general, to protect the superior characteristics of domestic animals, the assessment and preservation of diversity, and reconstruction of farm animal history are all necessary (Groeneveld et al., 2010). In this study, we analyzed different breed contributions to genetic diversity in order to establish conservation priority decisions for the indigenous pig populations around the Taihu Lake. From the perspective of entire populations around the Taihu Lake, their genetic distance is closer which indicates that these seven breeds originate from one breed. However, we found that these populations are somewhat differentiated (Figure 2), and show remarkable differences in genetic properties such as heterozygosity and allelic diversity, allowing to compute contributions to diversity and prioritization criteria for the different breeds under study.

We also calculated the average of the individual coancestry, the self-coancestry, and the average inbreeding together with the deviation from Hardy–Weinberg equilibrium (see Supplementary Table 4). For both inbreeding and coancestry estimates, the seven populations showed similar values, and average values of 0.74 and 0.70, respectively. In a randomly mating population, the values of coancestry and average inbreeding are supposed to coincide, whereas deviation from Hardy–Weinberg equilibrium can represent the extent of non-random mating. The deviations from Hardy–Weinberg equilibrium of the seven populations were all negative, which may suggest an excess of heterozygous genotypes exists within these populations which are currently under an active management in the conservation farms such as avoiding inbreeding. Overall, these metrics were similar in the seven Chinese indigenous pig populations. Among three types of genetic distance (DRij, DNij, and DSij), the estimates of Reynold’s genetic distance (DRij) were slightly larger than those of the other two [Nei-minimum genetic distance (DNij) and Nei-standard genetic distance (DSij)] (see Supplementary Table 5). Overall, the average value of coancestry was relatively high in magnitude, and all three genetic distances were relatively quite small. These results indicated that the seven pig populations originated from one larger population, which is consistent with the fact that all seven populations were considered “Taihu pigs” before 1974 in China (Zhang, 1987). Furthermore, the value of coancestry between MMS and SMS was the largest, and the three genetic distances between MMS and SMS were the smallest, which is consistent with the fact that MMS and SMS belong to one population called the Meishan pigs (MS).

Jiaxing black was the most differentiated breed, both following gene and allelic diversity criteria, and consistently contributed positively to the total diversity of the Taihu metapopulation (Figure 3). In addition, this breed retained the highest number of private alleles in the metapopulation, including loci with important functions to development, such as BMP5, suggesting that the JX breed has unique characteristics that make it worth preserving. However, the different effects of such identified loci still need to be validated either experimentally or through association studies.

Among the remaining breeds, SW and MI always ranked among the breeds contributing most to genic and allelic diversity (Figure 3). This is explained by their lower inbreeding coefficient compared to other breeds, as well as by their large level of differentiation, particularly measured by allelic distance. These results were also validated through a simulation approach where individuals from different subpopulations generate offspring and contribute randomly to a synthetic pool containing a mixture of individuals from different backgrounds (Figure 4). It is expected that when these contributions are maximized for genic diversity, both SW and MI contribute most to reach the goal of maximum expected heterozygosity in the newly generated population (i.e., the synthetic pool). On the contrary, the MMS breed is expected to have a lower contribution. The results from its contribution to genic diversity suggest that this breed contributes negatively to the metapopulation average diversity, given its high inbreeding coefficient (Figure 3A) and low genetic differentiation.

However, establishing priorities for MMS is not trivial, nor it is in fact for many of the remaining breeds. MMS is shown to have a negative overall contribution to genic diversity but positive for allelic diversity, and the opposite is shown for FJ. Thus, conservation priority decisions could differ based on one diversity criterion as opposed to the other. Moreover, various weights of within-breed and between-breed contributions to diversity can also lead to different results (Piyasatian and Kinghorn, 2003; Fabuel et al., 2004; Ollivier and Foulley, 2005). In consequence, it is also important to decide the most appropriate genetic criteria to establish conservation policies, and in the context of this study, this means weighing between genic and allelic diversity parameters. Both types of parameters are informative about the genetic diversity of populations. However, genic diversity is related to the expected heterozygosity, and thus maximizing it would prevent inbreeding and inbreeding depression (Ballou and Lacy, 1995). Higher heterozygosity also associates with higher additive variance, and thus a stronger response to selection (Falconer and Mackay, 1996). On the other hand, allelic diversity is more informative on population structure and bottlenecks and is a determinant of long-term response to selection and thus the adaptive potential of populations (Allendorf et al., 2013). This compromise between genic and allelic diversity has been previously explored by López-Cortegano et al. (2019b) using simulated populations, showing that, in general, allelic diversity was more robust to changes in the weights for within and between populations diversity. Besides, we observed that when the allelic diversity method was used to maximize diversity, it resulted in the highest overall allelic diversity as well as within-subpopulation genic diversity, thus maintaining a lower level of inbreeding in the metapopulation. In consequence, we advocate following allelic diversity criteria to help to make decisions for scenarios where priorities are obtained following genic and allelic diversity criteria conflict. Maximum allelic diversity also allows to preserve private alleles and functions that may be of future interest in breeding programs and thus should be promoted under long-term management programs for conservation.

Following the criterion on maximum allelic diversity, priorities for breeds from the Taihu lake region are given in Figure 3, with MI, SW, and EH clearly showing the higher contribution to diversity than the important breeds, highlighting the importance of preserving them, while FJ requires fewer conservation efforts. Regarding the Meishan breeds (MMS and SMS), MMS should be given priority compared to the SMS. This decision is not only supported by its higher contribution to the total metapopulation allele diversity but also by its higher number of private alleles, including one for the growth factor FGF14. The possible effect of private alleles found in SMS, however, should not be understated, and future studies should be directed to better understand the function and potential effects of loci such as these loci private alleles in traits of breeding importance, as future conservation priorities could be given by criteria based on genetic diversity, but also genetic functions to preserve.

Interestingly, seeing data for the MMS breed for instance seems that is the one contributing more genetic diversity (22%, Supplementary Table 1), but the metapopulation gains total gene diversity after its removal (Figure 3). This result may seem paradoxical, but we need to know that the total gene diversity in Supplementary Table 1 was calculated by 1-f times Ni/N, where Ni is the number of individuals in one breed. It can represent the effect of population size to some degree. When we remove the FJ population, the same phenomenon occurs in the total allele diversity. We also need to realize that the theoretical model we are considering is supposed to determine which populations contribute significantly to an indefinite pool of genes. Gene frequencies could become more equalized because of the removal of one subpopulation; therefore, the expected heterozygosity would be increased. There is another similar argument that suggests that the gene diversity of a meta-population would be increased if a subpopulation that includes mostly related individuals are eliminated and replaced by one with randomly chosen individuals.

## Data Availability Statement

The data used and analyzed in the current study are available *via* a public dataset (https://www.animalgenome.org/share/tmp/QXI1588224466.zip) and a private link (https://jbox.sjtu.edu.cn/l/XH2s6V).

## Ethics Statement

The animal study was reviewed and approved by Institutional Animal Care and Use Committee of Shanghai Jiao Tong University.

## Author Contributions

YP and QW supported and supervised this study. QZ analyzed the data and wrote the manuscript. EL-C helped analyze the data and revised the manuscript. ZZ and PM gave some important suggestions. All authors read and approved the manuscript.

## Conflict of Interest

The authors declare that the research was conducted in the absence of any commercial or financial relationships that could be construed as a potential conflict of interest.

## Supplementary Material

Supplementary Table 1 Contribution of each subpopulation to total gene diversity and allelic diversity.

Supplementary Table 2 Loss (+) or gain (−) of gene diversity after removal of each subpopulation.

Supplementary Table 3 Loss (+) or gain (−) of allelic diversity after removal of each subpopulation.

Supplementary Table 4 Parameters of subpopulations.

Supplementary Table 5 Parameters between subpopulations.

Supplementary Table 6 Private loci for each breed.
