# Supplementary material for: Conservation Priorities Analysis of Chinese Indigenous Pig Breeds in the Taihu Lake Region
Source: Front Genet. 2021 Mar 3;12:558873. doi: 10.3389/fgene.2021.558873 (PMC7966724; doi:10.3389/fgene.2021.558873)
Supplement: Supplementary Table 1 — Contribution of each subpopulation to total gene diversity and allelic diversity. [file Table_1.DOCX]

**Supplementary Material**

**Table S1** Contribution of each subpopulation to total gene diversity and allelic diversity

| **Sub** | **WI^g^** | **BI^g^** | WS^g^ | **BS^g^** | **T^g^** | **W^a^** | **B^a^** | **T^a^** |
| --- | --- | --- | --- | --- | --- | --- | --- | --- |
| **FJ** | 0.011  (7%) | 0.006  (6%) | 0.017  (7%) | 0.004  (11%) | 0.021  (14%) | 0.119  (13%) | 0.012  (16%) | 0.131  (14%) |
| **MI** | 0.012  (8%) | 0.009  (9%) | 0.021  (8%) | 0.003  (8%) | 0.025  (17%) | 0.131  (15%) | 0.010  (14%) | 0.141  (15%) |
| **EH** | 0.013  (9%) | 0.012  (12%) | 0.025  (10%) | 0.003  (8%) | 0.028  (10%) | 0.131  (15%) | 0.009  (12%) | 0.141  (15%) |
| **SW** | 0.030  (20%) | 0.015  (14%) | 0.044  (17%) | 0.005  (14%) | 0.050  (17%) | 0.131  (15%) | 0.010  (14%) | 0.141  (15%) |
| **JX** | 0.027  (18%) | 0.022  (21%) | 0.049  (19%) | 0.010  (27%) | 0.059  (20%) | 0.123  (14%) | 0.012  (16%) | 0.135  (14%) |
| **MMS** | 0.033  (22%) | 0.023  (22%) | 0.056  (22%) | 0.007  (20%) | 0.063  (22%) | 0.130  (15%) | 0.010  (14%) | 0.140  (15%) |
| **SMS** | 0.025  (17%) | 0.017  (16%) | 0.042  (16%) | 0.006  (16%) | 0.049  (17%) | 0.125  (14%) | 0.011  (15%) | 0.136  (14%) |
| **Total** | 0.151 | 0.104 | 0.255 | 0.037 | 0.293 | 0.890 | 0.074 | 0.964 |

Note: WI^g^: gene diversity within individuals; BI^g^: gene diversity between individuals; WS^g^: gene diversity within subpopulations; BS^g^: gene diversity between subpopulations; T^g^: total gene diversity; W^a^: within subpopulations; B^a^: between subpopulations; T^a^: total allelic diversity; FJ: Fengjing, MI: Mizhu; EH: Erhualian; SW: Shawutou; JX: Jiaxing black; MMS: Middle Meishan; SMS: Small Meishan.

**Table S2** Total gene diversity after removing subpopulation i and loss (+) or gain (-) of diversity after removal of each subpopulation (%)

| **Subpop.removed** | **GD_T\i** | **GD_WI** | **GD_BI** | **GD_BS** | **GD_T** |
| --- | --- | --- | --- | --- | --- |
| **FJ** | 0.291 | 0.208 | -0.677 | 0.907 | 0.438 |
| **MI** | 0.291 | -0.015 | 0.254 | 0.396 | 0.635 |
| **EH** | 0.291 | -0.503 | 0.866 | 0.154 | 0.517 |
| **SW** | 0.290 | 2.175 | -0.887 | -0.381 | 0.906 |
| **JX** | 0.290 | -1.685 | 0.414 | 2.032 | 0.762 |
| **MMS** | 0.295 | -0.110 | 0.185 | -0.790 | -0.716 |
| **SMS** | 0.292 | -0.139 | -0.147 | 0.491 | 0.205 |

Note: GD_T\i: total gene diversity after removing subpopulation i; GD_WI: gene diversity within individuals; GD_BI: gene diversity between individuals; GD_BS: gene diversity between subpopulations; GD_T: the total loss or gain gene diversity;

**Table S3** Total allelic diversity after removing subpopulation i and loss (+) or gain (-) of diversity after removal of each subpopulation (%)

| **Subpop.removed** | **AD_T\i** | **A_S_** | **D_A_** | **A_T_** |
| --- | --- | --- | --- | --- |
| **FJ** | 0.968 | -0.995 | 0.543 | -0.4511 |
| **MI** | 0.960 | 0.487 | -0.080 | 0.407 |
| **EH** | 0.961 | 0.508 | -0.188 | 0.319 |
| **SW** | 0.960 | 0.460 | -0.128 | 0.332 |
| **JX** | 0.963 | -0.485 | 0.586 | 0.101 |
| **MMS** | 0.961 | 0.279 | 0.021 | 0.301 |
| **SMS** | 0.963 | -0.255 | 0.315 | 0.061 |

Note: AD_T\i: total allelic diversity after removing subpopulation i; A_S_: allelic diversity within subpopulations; D_A_: allelic diversity between subpopulations; A_T_: the total loss or gain allelic diversity;

**Table S4** Parameters of subpopulations

| Subpopulation | ***f_ii_*** | ***s_i_*** | ***F_i_*** | ***d_ii_*** | ***G_i_*** | ***a_i_*** |
| --- | --- | --- | --- | --- | --- | --- |
| **FJ** | 0.76 | 0.84 | 0.68 | 0.08 | 0.33 | -0.34 |
| **MI** | 0.74 | 0.85 | 0.70 | 0.11 | 0.43 | -0.15 |
| **EH** | 0.73 | 0.86 | 0.73 | 0.13 | 0.48 | -0.03 |
| **SW** | 0.73 | 0.82 | 0.63 | 0.09 | 0.33 | -0.34 |
| **JX** | 0.76 | 0.87 | 0.74 | 0.11 | 0.45 | -0.10 |
| MMS | 0.74 | 0.85 | 0.70 | 0.11 | 0.41 | -0.17 |
| **SMS** | 0.75 | 0.85 | 0.70 | 0.10 | 0.41 | -0.19 |
| **Average** | 0.74 | 0.85 | 0.70 | 0.10 | 0.41 | -0.19 |

Note: *f_ii_*: coancestry; *s_i_*: self-coancestry; *F_i_*: inbreeding; *d_ii_*: distance; *G_i_*: proportion of diversity between individuals of subpopulation; *a_i_*: deviation from Hardy–Weinberg equilibrium. FJ: Fengjing, MI: Mizhu; EH: Erhualian; SW: Shawutou; JX: Jiaxing black; MMS: Middle Meishan; SMS: Small Meishan;

**Table S5** Parameters between subpopulations

| **Subpopulation** | ***f_ij_*** | ***DR_ij_*** | ***DN_ij_*** | ***DS_ij_*** |
| --- | --- | --- | --- | --- |
| **FJ - MI** | 0.685 | 0.204 | 0.064 | 0.090 |
| **FJ - EH** | 0.695 | 0.174 | 0.053 | 0.073 |
| **FJ – SW** | 0.701 | 0.145 | 0.043 | 0.060 |
| **FJ - JX** | 0.704 | 0.192 | 0.057 | 0.078 |
| **FJ - MMS** | 0.699 | 0.181 | 0.055 | 0.075 |
| **FJ - SMS** | 0.690 | 0.213 | 0.066 | 0.091 |
| **MI - EH** | 0.708 | 0.095 | 0.028 | 0.038 |
| **MI - SW** | 0.689 | 0.137 | 0.043 | 0.060 |
| **MI – JX** | 0.690 | 0.188 | 0.058 | 0.081 |
| **MI – MMS** | 0.701 | 0.133 | 0.040 | 0.055 |
| **MI - SMS** | 0.701 | 0.141 | 0.042 | 0.059 |
| **EH - SW** | 0.695 | 0.115 | 0.035 | 0.049 |
| **EH – JX** | 0.695 | 0.169 | 0.052 | 0.072 |
| **EH – MMS** | 0.704 | 0.121 | 0.036 | 0.050 |
| **EH - SMS** | 0.698 | 0.146 | 0.044 | 0.061 |
| **SW - JX** | 0.694 | 0.157 | 0.048 | 0.067 |
| **SW - MMS** | 0.708 | 0.091 | 0.027 | 0.037 |
| **SW – SMS** | 0.700 | 0.126 | 0.038 | 0.052 |
| **JX - MMS** | 0.701 | 0.169 | 0.050 | 0.069 |
| **JX - SMS** | 0.693 | 0.198 | 0.061 | 0.084 |
| **MMS - SMS** | 0.718 | 0.102 | 0.029 | 0.039 |

Note: *f_ij_*: values of coancestry; *DR_ij_*: Reynold's genetic distance; *DN_ij_*: Nei-minimum genetic distance; *DS_ij_*: Nei-standard genetic distance. FJ: Fengjing, MI: Mizhu; EH: Erhualian; SW: Shawutou; JX: Jiaxing black; MMS: Middle Meishan; SMS: Small Meishan;
